# Common Sense in Choice: The Effect of Sensory Modality on Neural Value Representations

**DOI:** 10.1523/ENEURO.0346-17.2018

**Published:** 2018-04-04

**Authors:** Anastasia Shuster, Dino J. Levy

**Affiliations:** 1 Coller School of Management, Tel Aviv University, Tel Aviv 6997801, Israel; 2 Sagol School of Neuroscience, Tel Aviv University, Tel Aviv 6997801, Israel

**Keywords:** decision making, fMRI, sensory systems, value

## Abstract

Although it is well established that the ventromedial prefrontal cortex (vmPFC) represents value using a common currency across categories of rewards, it is unknown whether the vmPFC represents value irrespective of the sensory modality in which alternatives are presented. In the current study, male and female human subjects completed a decision-making task while their neural activity was recorded using functional magnetic resonance imaging. On each trial, subjects chose between a safe alternative and a lottery, which was presented visually or aurally. A univariate conjunction analysis revealed that the anterior portion of the vmPFC tracks subjective value (SV) irrespective of the sensory modality. Using a novel cross-modality multivariate classifier, we were able to decode auditory value based on visual trials and vice versa. In addition, we found that the visual and auditory sensory cortices, which were identified using functional localizers, are also sensitive to the value of stimuli, albeit in a modality-specific manner. Whereas both primary and higher-order auditory cortices represented auditory SV (aSV), only a higher-order visual area represented visual SV (vSV). These findings expand our understanding of the common currency network of the brain and shed a new light on the interplay between sensory and value information processing.

## Significance Statement

Whether auditory information has the same impact on the value system as visual information is unknown. This is striking, given that we live in a multimodal world, and decision making relies on evidence from all of our senses. It is well-known that the ventromedial prefrontal cortex (vmPFC) represents value in an abstract and common manner, which allows comparison between different options. We examined whether this representation is sensitive to the sensory modality in which options are presented. Using fMRI and a risk-evaluating task, we show that whereas sensory cortices represent value of options in a modality-specific way, the vmPFC represents value irrespective of sensory modality. This brings our understanding of the neural value system closer to choices in the real world.

## Introduction

Throughout our daily life, the brain computes and assigns values for items or concepts it comes across. These value labels allow us to compare alternatives and decide which is preferable, for example, staying in bed for ten more minutes or getting up to go to work. Previous studies have identified a neural network which represents value in an abstract way (Levy and Glimcher, 2012; Clithero and Rangel, 2014). This network, termed the common currency network, is composed mainly of the ventromedial prefrontal cortex (vmPFC) and the ventral striatum (vStr; Levy and Glimcher, 2012; Bartra et al., 2013). Both these regions track and represent reward values irrespective of its identity or type, ranging from tangibles such as DVDs (Levy et al., 2011) and snack foods (Plassmann et al., 2007; Levy and Glimcher, 2011; Pogoda et al., 2015), to pastime activities (Gross et al., 2014), attractive faces (O’Doherty et al., 2003), and social interactions (Lin et al., 2012; Ruff and Fehr, 2014). However, such representations should allow us to compare not only across categories, but also between value of heard information, like footsteps of a loved one, and value of things we see, such as their smile.

Only a handful of experiments examining the properties of this network employed sensory modalities other than visual (Kühn and Gallinat, 2012). Studies of the neural value representations of odors (O’Doherty et al., 2000; Anderson et al., 2003; Grabenhorst et al., 2007; Howard et al., 2015), tastes (Doherty et al., 2002; McCabe and Rolls, 2007), and their combination (De Araujo et al., 2003) showed a correlation between the activity of the medial orbitofrontal cortex and ratings of subjective pleasantness. One study examined the representation of beauty in the brain by comparing neural activity in response to listening to music and looking at paintings. The authors found that both conditions activate the vmPFC as a function of how beautiful a stimulus is (Ishizu and Zeki, 2011). However, subjects did not make actual choices between options, and it is not clear if beauty ratings are similar to value-based choices. Therefore, it remains unknown whether the common currency network represents subjective value (SV) irrespective of sensory modality. In other words, just how common is the common currency network?

A complement question is the nature of value representation in sensory cortices. In the visual domain, value modulation was observed in both early (Serences, 2008; Zeki and Stutters, 2012) and higher areas (Chatterjee et al., 2009), such that neural activity was correlated with rewarding properties of visual stimuli. There is evidence for value modulation in the auditory domain as well (Thiel et al., 2002; Weinberger, 2007; Brosch et al., 2011; Puschmann et al., 2013), suggesting that associating a specific tone with appetitive or aversive consequences can alter the neural activity of the auditory cortex. Furthermore, neural correlates of pleasure derived from music appear in both vStr and superior temporal gyrus, where the auditory cortex lies (Mueller et al., 2015), and the functional coupling between them increases as music’s reward value increases (Salimpoor et al., 2013). Taken together, these findings suggest that sensory cortices are influenced by value. However, whether this influence is modality-specific or cross-modal is unclear. Furthermore, to our knowledge, no study has examined the auditory cortex during visual decision making or vice versa. Finally, it is unknown where within the sensory cortex such a representation resides.

To address these questions, we conducted a neuroimaging study using a standard risk task (Holt and Laury, 2002, 2005; Levy and Glimcher, 2011), in which subjects performed a series of choices between a risky and a safe alternative. Importantly, the alternatives were presented either visually or aurally. Using this task, we identified regions of the brain representing SV as a function of the modality of presentation, as well as brain areas tracking SV irrespective of the sensory modality. We then used cross-modality classifiers to examine whether this representation is truly generic, such that training a classifier on one modality can distinguish values in the other. Two potential outcomes exist: one, in line with evidence that the vmPFC represents stimuli of various modalities, the common-currency network could represent visually-presented rewards similarly or identically as the same rewards presented aurally. Alternatively, since visual and auditory information are not equivalent in terms of their neural representation, the brain is biased toward visual information, both anatomically (Zeki, 1993) and functionally (e.g., Bigelow and Poremba, 2014), it is possible that visually-presented rewards will yield a different representation within the value system, compared to the aurally-presented rewards.

## Materials and Methods

### Participants

Forty-three healthy subjects participated in the study (21 females, mean age 25, 20–43). Subjects gave informed written consent before participating in the study, which was approved by the local ethics committee at Tel Aviv University. All subjects completed at least one behavioral session. Of them, 40 completed the second behavioral session (one dropped out, and two were excluded due to random choice behavior). Of them, 26 participated in the neuroimaging session: six subjects opted out, two did not complete a full scanning session due to technical problems (one was accidently scanned using the wrong protocol, and one had troubles using the MR-compatible glasses), five subjects were not called back due to inconsistent risk preferences, and one due to random choice behavior in the second session.

### Experimental design

#### Risk-evaluating task

**Figure 1.**
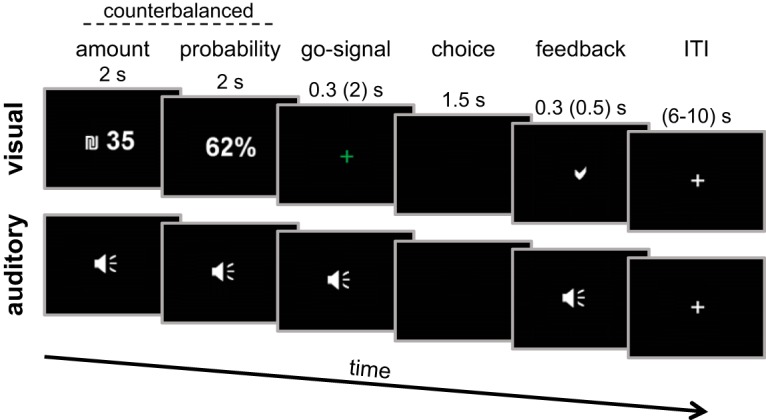
Experimental procedure: trial timeline. On each trial, subjects saw or heard a lottery, a winning probability followed by an amount of money (the order of the presentation of the amount and probabilities were counterbalanced across trials). After a short go-signal, subjects chose between the lottery and a sure amount of money, which was always 10 NIS and was not presented on each trial. Numbers on top represent the duration in seconds in the behavioral sessions. In brackets are the durations in the fMRI experiment. s, seconds; ITI, intertrial interval.

On each trial, subjects chose between a presented *lottery* and a certain amount of money (a *reference* option). The lottery consisted of an amount of money [10, 35, 45, 50, or 75 New Israeli Shekels (NIS); 1 NIS is ∼0.25 USD] and a chance of winning it (15%, 30%, 45%, 62%, or 80%), presented consecutively. The presentation order was counterbalanced across trials. The reference option was always a certain amount of 10 NIS, and it was not presented to subjects during the trials, only in the instructions stage, and they were reminded about it at the beginning of each block. Each lottery was presented either visually or aurally (Fig. 1). On visual trials, the amount and probability appeared as white text on a black background for 2 s each. Next, a green fixation-cross appeared for 300 ms (2 s in the fMRI session), after which subjects indicated their choice (lottery or reference) by clicking on the right or left buttons of a computer mouse. The buttons’ encoding remained constant throughout trials, blocks and sessions for each subject, but was counterbalanced between subjects. The time window to indicate a choice was 1.5 s long. Next, a feedback appeared on screen, a check mark in cases when the subject made a choice, and a text reading “no choice was made” otherwise. On auditory trials, subjects heard male voice recordings of the phrases “## shekalim” and “at ## percentage” via headphones. As in visual trials, amount and probability were presented for 2 s each, followed by a beeping sound (similar to the green fixation-cross in the visual trials), signaling subjects to choose. A feedback for response was either another beep in case the subject made a choice, or a buzzer sound in case the subject failed to respond within the allotted time.

#### Stimuli

In the behavioral sessions, we used all 25 lottery-options that can be composed using the five amounts and five probabilities. In the fMRI session, we used a subset of 13 options, sampling the center of the payoff matrix and some of the corners: 10 NIS at 15%, 10 NIS at 80%, 35 NIS at 30%, 35 NIS at 45%, 35 NIS at 62%, 45 NIS at 35%, 45 NIS at 45%, 45 NIS at 62%, 50 NIS at 35%, 50 NIS at 45%, 50 NIS at 62%, 75 NIS at 45%, and 75 NIS at 80%. For the auditory lotteries, we used a Hebrew text-to-speech software (Alma Reader, Kolpics), to create audio files of the amounts and probabilities read out-loud. Each stimulus lasted ∼2 s. In the fMRI session, the stimuli were delivered using S14 in-ear headphones by Sensimetrics lcd, and the volume amplitude was adjusted manually per subject to ensure that the auditory task is delivered in a clean and well-balanced manner and overcomes the MR background noise (∼75 db).

#### Behavioral sessions

Before starting the first behavioral session, subjects gave written consent, read the instructions and filled out a short demographics questionnaire. Next, they underwent a short training session, consisting of five auditory trials and five visual trials. On successful completion of the training, subjects preformed the risk-evaluating task. The task was composed of 12 blocks, six visual and six auditory, presented at a random order. Each block consisted of 25 trials presented at a random order. At the end of the task, one trial was randomly selected, implemented and paid out to the subject in addition to the participation fee. After completing the first session, we assessed each subject’s risk-preference (for details, see below, Risk-preference estimation). Subjects with behavior that we were unable to fit with a utility function (due to random choices) were considered as outliers and were not asked to return. The other subjects were called in for a second behavioral session, in which they performed the task again. The average time elapsed between the first and the second session was 13.025 d (5–61). We then calculated subjects’ risk-preference based on the second session, and subjects with stable scores (a difference smaller than 0.25 in their estimated risk-preference parameter) were called back for the final fMRI session. We chose the 0.25 threshold based on the behavior of the first ten participants in our study; their average difference across sessions was 0.1, with a standard deviation of 0.135. Therefore, any subject with a difference of more than one SD of the mean was discontinued from the study. The average elapsed time between the second behavioral session and the fMRI session was 44.42 d (5–311). Note, that the first two subjects in the experiment had an extremely long duration between behavioral sessions and fMRI scans. When disregarding them, the average time between the second session and the fMRI session dropped to 24.45 d (5–94). Notwithstanding, both subjects’ risk-preference scores remained stable between the three time points, with changes in their fitted risk preference parameter (α) < 0.12.

#### fMRI session

In the fMRI session, subjects performed the risk-evaluating task while being scanned. The task was identical to the task used in the behavioral sessions, except for the addition of an intertrial interval to account for the hemodynamic delay (mean duration 8 s, jittered between 6, 8, and 10 s). Each run consisted of three repetitions of the 13 possible trials (a total of 39 trials per run) for a given sensory modality, and each subject completed a total of four functional runs. The runs were randomly ordered across the session. After completing the main task, we obtained an anatomic scan for each subject. Lastly, each subject completed two functional localizers, one visual and one auditory, to identify individual loci of sensory activations, visual and auditory (for details, see below, Functional localizers). At the end of the fMRI session, one trial was selected at random and paid out to the subject, in addition to the participation fee.

#### Functional localizers

For the visual localizer, we used two visual categories: objects (black-and-white images) and scrambled objects (the same objects broken into pixels and scrambled into nonrecognizable images). The localizer consisted of 21 blocks. The duration of each block was 16 s, and blocks were presented in a pseudo-random order. Out of the 21 blocks, eight were objects blocks, eight were scrambled objects, and the remaining five blocks were interleaved between the other blocks, consisting of a blank-screen which served as a baseline. Within each block, 20 images were presented for 800 ms per image. To make sure that subjects payed attention to the localizer stimuli presented on the screen, at the beginning of each run we presented two images (one object and one scrambled) from the pool of stimuli and instructed subjects to memorize both images and press a key whenever they appeared on screen during the run.

To locate auditory sensitive regions, we used a localizer with auditory stimuli of three categories: silence (baseline), non-vocal sounds taken from The Voice Neurocognition Laboratory in University of Glasgow (e.g., birds chirping, cars honking, etc.; Pernet et al., 2015), and sequences of beeps. The auditory localizer consisted of 21 blocks. The duration of each block was 8 s and blocks were presented in a pseudo-random order. Eight blocks were non-vocal, eight were beeps, and the remaining five were silence serving as baseline. As in the visual localizer, we asked subjects to memorize a particular sound beforehand, and to press a key whenever it appeared in the run.

### Image acquisition

Scanning was performed at the Strauss Neuroimaging Center at Tel Aviv University, using a 3T Siemens Prisma scanner with a 64-channel Siemens head coil. Anatomic images were acquired using MPRAGE, which comprised 208 1-mm-thick axial slices at an orientation of −30° to the AC–PC plane. To measure blood oxygen level-dependent (BOLD) changes in brain activity during the risk-evaluating task, a T2*-weighted functional multi-echo EPI pulse sequence was used [TR = 2 s; TE = 30 ms; flip angle = 90°; matrix = 74 × 74; field of view (FOV) = 222 mm; slice thickness = 3 mm]; 33 axial (−30° tilt) 3-mm slices with no interslice gap were acquired in ascending interleaved order. To measure neural activity during the functional localizers, a multi-band EPI sequence was used (TR = 2 s, TE = 30 ms; flip angle = 90°; matrix = 112 × 112; FOV = 224 mm; slice thickness = 2 mm); 58 axial (−30° tilt) 3-mm slices without gaps were acquired in an ascending interleaved order.

### Image analysis

BrainVoyager QX (Brain Innovation, RRID:SCR_006660) was used for image analysis, with additional analyses performed in Matlab (MathWorks, RRID:SCR_001622). Functional images were sinc-interpolated in time to adjust for staggered slice acquisition, corrected for any head movement by realigning all volumes to the first volume of the scanning session using six-parameter rigid body transformations, and de-trended and high-pass filtered to remove low-frequency drift in the fMRI signal. Data were also spatially smoothed with a Gaussian kernel of 4 mm (full-width at half-maximum). Note that for the multivariate analysis, we used the nonsmoothed data. Runs in which a subject moved >3 mm were removed from any further analyses (a total of three runs were removed). Images were then coregistered with each subject’s high-resolution anatomic scan and normalized using the Montreal Neurologic Institute (MNI) template. All spatial transformations of the functional data used trilinear interpolation.

### Risk-preference estimation

We used random utility theory to derive the subject-specific estimated SV for each modality. We pooled the choice data from all three sessions (two behavioral and one fMRI) and separated it into visual and auditory trials. For each subject, we modeled the utility functions for each sensory modality separately as power functions having the form

$$EU\left(X,p\right)=p\times X^{\alpha_{sm}}+(1-p)\times 0^{\alpha_{sm}}$$

where p is the probability for an option to yield a reward (in the reference alternative this is equal to 1, and in the lottery alternative it varies between trials), X is the amount (in NIS) of the offered reward, and $\alpha_{sm}$ is the free parameter representing the subject-specific (s) modality-specific (m) attitude toward risk. With a power utility function, a value of α = 1 denotes risk-neutrality, a value of α > 1 represents a risk-seeking individual with a convex utility function, and an α < 1 represents a risk-averse individual with a concave utility function. We selected this particular equation to fit the utility for its simplicity, minimal assumptions, having only one free parameter, and its ability to predict choice behavior.

Using maximum likelihood estimation (MLE), we fitted the choice data of each modality to a single logistic function of the form

$$P_{L}=\frac{1}{1+e^{\beta_{sm}\times ({EU}_{L}-{EU}_{R})}}$$

where $P_{L}$ is the probability that the subject chose the lottery option, ${EU}_{L}$ and ${EU}_{R}$ are the expected utility for the lottery and reference option, respectively, and $\beta_{sm}$ is the slope of the logistic function, which is the second subject-specific modality-specific free parameter. This analysis produced a fitted risk-preference parameter ($\alpha_{sm}$) and a slope parameter ($\beta_{sm}$) for each sensory modality. It thus specified a utility function (or equivalently, a SV function) for each modality for each subject that could account for the trade-offs between risk and reward that we observed in our subjects.

To use the SV as a parametric regressor in our analysis, we calculated for each subject in each sensory modality the SV of a trial *t* (${SV}_{t}$), defined as the probability to win multiplied by the amount to the power of subject’s risk preference:

$${SV}_{t}={probability}_{t}\;\text{×}\;{{amount}_{t}}^{{\text{α}}_{sm}}$$

To examine the behavioral data for differences in risk preferences between sensory modalities, we averaged the risk parameters across subjects for a given sensory modality and used the nonparametric rank test.

To ensure that differences in reaction times (RTs) cannot explain the neural response to different levels of value, we conducted two tests. One, we created a linear regression of RTs and SVs of each trial of the 26 participants in the fMRI session and clustered the errors by subject. A significant coefficient would indicate that any neural results might by due to an effect of elapsed time on the trial. Second, we correlated RTs to visual lotteries with RTs to auditory lotteries. To do so, we first arranged the data to have the same number of samples, such that if a subject missed a trial in one modality, we omitted a trial of the same lottery from the other modality. Then we sorted the RTs according to the lotteries, to make them comparable across modalities. Since the neural classification analysis is subject specific, we correlated each subject’s auditory RTs with visual RTs.

### Statistical analysis

#### Whole-brain analysis of SV

To identify the neural correlates of auditory SV (*aSV*) and of visual SV (*vSV*), we created a general linear model (GLM) with 11 predictors. The first two predictors contained the trial-by-trial SVs, separated by modality. Note, that these values relate to the SV of the lottery presented on the screen, irrespective of subject’s choice. These values were entered at the first two TRs of each trial, normalized and convolved with the canonical hemodynamic response function (HRF). Another two dummy predictors represented trial identity, also separated into modalities and convolved with the HRF (*aStick* and *vStick*). The additional seven predictors consisted of six nuisance predictors, obtained from the motion-correction stage, and a constant. Results were corrected for multiple comparisons using FDR correction.

Since the representation of value might be linked to choice, such that it encodes the value of the chosen alternative and not the offer (Raghuraman and Padoa-Schioppa, 2014), we conducted an additional GLM, modeling chosen SV instead of the presented SV. In this GLM the trial-by-trial SV was equal to the lottery SV in case the subject chose the lottery, and to the reference SV in case the subject chose the reference option. All reference trials were equal to ${SV}_{reference}=1\text{×}10^{{\text{α}}_{sm}}$. We modeled missed trials as 0. Then, we computed a Pearson correlation between the chosen-SV and the presented-SV regressors to examine possible differences between the two models, applied the chosen-SV model to the neural data and compared it to the presented-SV results.

#### Region of interest (ROI) analysis in sensory cortices

To identify value modulation in sensory areas, we first pinpointed eight ROIs for each subject (when possible), based on the functional localizers’ data. Primary visual cortices (both left and right) were defined as the peak activity when using the contrast scrambled objects > objects, at a significance level of *z* = 4. Similarly, we identified higher-order visual cortices using the opposite contrast (objects > scrambled objects) at the same significance threshold. Primary auditory cortices were defined as the peak activity when using the contrast beeps > silence at a significance level of *z* = 4, and higher-order auditory cortices were defined using the contrast non-vocal sounds > beeps, at a slightly lower statistical threshold of *z* = 3, due to overall reduced activations. We then conducted the same GLM mentioned above and correlated SV with BOLD activity extracted from each of the eight subject-specific sensory ROIs. To examine if a sensory ROI is representing SV, we conducted two tests: one, we compared the β-values of *aSV* and *vSV* of each ROI to zero, using one-sample two-tailed *t* tests. Second, to test for the specificity of the value representation, we directly compared *aSV* to *vSV* using one-tailed paired-samples *t* tests. Additionally, to be certain that the ROIs are indeed sensory, we compared the β-values of the trial identity dummy variables (*aStick* and *vStick*) to zero.

As the auditory localizer is less commonly used than the visual localizer, we wished to replicate any finding related to it by implementing an alternative method of defining it. We achieved this by using the web-based tool NeuroSynth (Yarkoni, 2014), that allows to create neural maps based on meta-analyses of the literature. We downloaded two maps, corresponding to “Heschl gyrus” and “planum temporale.” Both maps were set to *z* = 10.5 to identify the most central region. Then, we applied the same four-predictor GLM, extracted β-values from right and left ROIs and repeated one-sample (against zero) and paired-samples (*aSV* > *vSV*) *t* tests. All of the reported results were corrected for multiple comparisons using Bonferroni correction.

### Multivoxel pattern analysis (MVPA)

Our main objective was to further strengthen our GLM findings that voxels in the vmPFC represent value on a common scale. To do so, we used a cross-modality classification algorithm, which allowed us to test the similarity of neural representation between conditions, visual and auditory. We used a MVPA ROI searchlight approach (Kriegeskorte et al., 2006) to determine voxels that exhibit a significant difference in activation between low- and high-value trials, evaluating their value representation property. Furthermore, the cross-modality approach allowed us to determine voxels that are not only sensitive to value, but also not sensitive to sensory modality. Thus, the searchlight analysis enables us to define subregions of the vmPFC that represent modality-free value. To do so, we first determined low- and high-SV trials (*lSV* and *hSV*) for each subject using a median split. We restricted our analysis to the vmPFC, which was defined using an ROI from a meta-analysis of value representation (Bartra et al., 2013). The unsmoothed BOLD signal of each voxel in the ROI was *z*-scored, to account for signal intensity variations across runs. We then extracted the signal at the fourth TR (6 s) after stimulus onset. For each center-voxel, data of the 24 closest voxels (in Euclidean distance) during *lSV* and *hSV* trials was used as input to the classifier. We used a Matlab implementation of a support vector machine (SVM) classifier (Chang and Lin, 2011; RRID:SCR_010243) to classify *lSV* from *hSV*. To obtain a prediction score for the center-voxel, a repetitive leave-2-out cross-validation analysis was performed, in which we trained the SVM to classify *lSV* and *hSV* trials of one sensory modality but tested it on trials from the other sensory modality. One trial of each condition (*lSV* and *hSV*) from the opposite modality was used as the test-set and all remaining trials of the trained modality were used as the training. The model’s prediction score could vary between 0% (unsuccessful classification of both test trials), 50% (one successful), and 100% (both successful). The overall classification for each center-voxel is an average of the prediction accuracy score over the total number of iterations (100). This process was repeated for each voxel within the ROI, for each subject and each modality separately. To test for significance, we used a nonparametric permutation test, in which the labels for the high- and low-value trials were randomly shuffled on each leave-2-out iteration, and a prediction score was computed. On each iteration, we used 100 permutations of the labels, and averaged over the iterations. We ran the process in two modes of classification, train-on-auditory and test-on-visual, and train-on-visual and test-on-auditory. Thus, each voxel was assigned two “real” (i.e., unshuffled) scores, and 200 shuffled scores. We then averaged the classification results across the two modes of classification, yielding one real and 100 shuffled scores. Voxels were considered significant if their real performance exceeded 95% of the shuffled accuracy scores. To combine the results of all 26 subjects together, we created a probability map of the significant voxels across subjects from the classification step. For presentation purposes, we present only voxels that had a significant classification in at least 75% of subjects.

To test the robustness of the vmPFC-only result, we performed an additional whole-brain searchlight cross-modality classification analysis. To do so, we defined a gray-matter mask, and extracted the BOLD data from each voxel, as in the vmPFC-only analysis. The searchlight size was adapted for the bigger mask, to consist 125 voxels. All other parameters of the classifier remained identical. We then computed a probability map of significant voxels across subjects, thresholded at 75% of subjects and restricted cluster size to be at least 15 continuous voxels.

## Results

### Behavior

**Figure 2.**
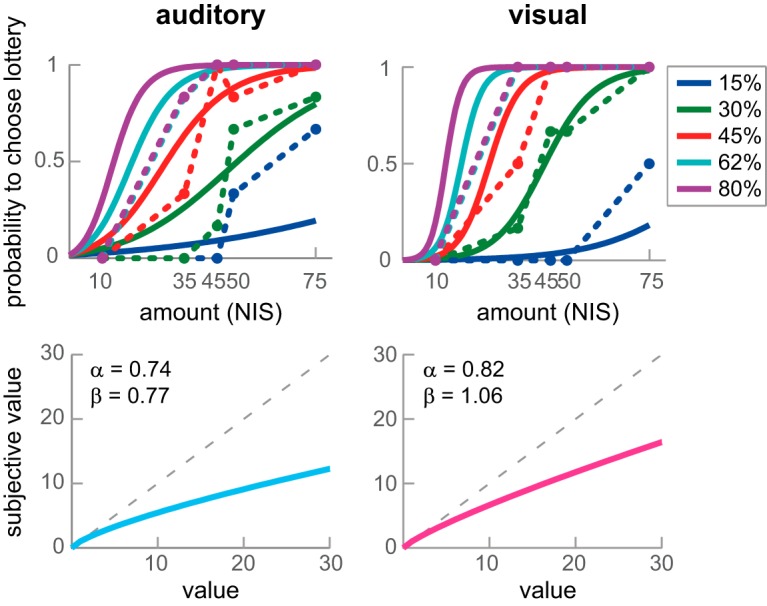
Example subject: choice behavior, fitted logits, and utility functions. Left panels, Auditory data. Right panels, Visual data. Top panel, Actual choice data (dashed lines) and best-fitted logits (solid lines). The graphs describe the propensity to choose the lottery option as a function of the monetary amount of the lottery option. The different colors represent the five different winning probabilities of the lottery option. Bottom panel, Utility functions (solid lines) derived from the choice data and the estimated risk preference parameters (α and β) for the example subject for each modality. The utility function translates objective reward magnitude to the observed SV. The dashed line represents the unity line. This subject is characterized by a mild risk aversion (α < 1).

Subjects performed a risk-evaluating task inside an fMRI scanner (Fig. 1). On each trial subjects chose between a safe and a risky alternative. The safe alternative was a certain amount of money (the reference option, 10 NIS). The risky alternative was a lottery, some probability to win some amount of money. The probabilities and amounts varied across trials. Importantly, we presented the amount and probability information either visually on a computer screen (the visual condition) or aurally via headphones (the auditory condition). We first calculated each subject’s risk-preference in each modality separately, by fitting a logistic function to their choice behavior, using a MLE process with two free parameters, α and β (see Materials and Methods). The α parameter represents subjects’ attitude toward risk, with scores under 1 representing risk-aversion, and scores above 1 representing risk-seeking. The β-parameter is the inverse temperature, or the slope of the logistic function, and it represents the level of noise in choices. We next constructed each subject’s utility function separately for each modality, based on their own estimated risk-preferences. Figure 2 depicts results from an example subject. Note, that we report data and analyses conducted on the 26 subjects that performed both behavioral sessions and the fMRI session.

**Figure 3.**
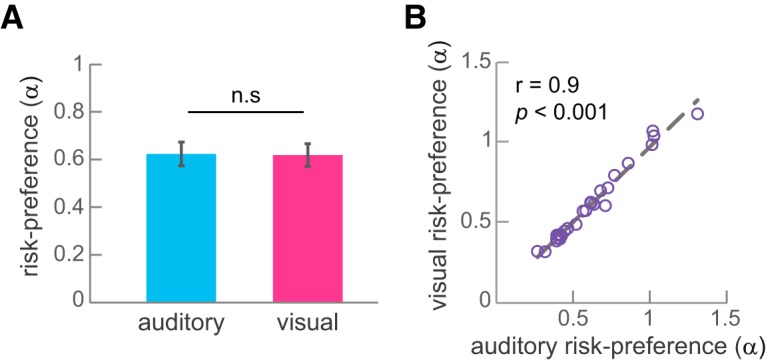
Risk preferences across sensory modalities; *n* = 26. ***A***, Mean estimated risk-preference across subjects (α-parameter). Error bars indicate SEM. ***B***, Within-subject Spearman’s correlation between the estimated risk preference parameter in the auditory and visual conditions. Each circle represents a subject.

We first examined whether there is a difference between risk preferences across sensory modalities. As can be seen in Figure 3*A*, in the auditory condition the average risk preference was 0.62, with scores ranging from 0.26 for the most risk-averse subject to the most risk-seeking subject with a score of 1.31. In the visual condition the average risk preference was 0.61, with scores ranging from 0.31 to 1.17. We did not find a significant difference between α_auditory_ and α_visual_ (*n* = 26, *Z* = −0.045, *p* = 0.96, Wilcoxon rank-sum test). This suggests that on average subjects display similar levels of risk preferences irrespective if the choice is presented visually or aurally. The slopes of the logistic functions (the β-parameter) did not differ between modalities as well (*n* = 26, *z* = 0, *p* = 1).

We next examined whether risk preferences across sensory modalities are correlated within subjects. As can be seen in Figure 3*B*, risk preferences across sensory modalities are highly correlated within subjects (Spearman R = 0.97, *p* < 0.001). Subjects show a high correlation of the slopes of the logistic function as well (Spearman R = 0.95, *p* < 0.001). This suggests that subjects’ attitudes toward risk are preserved across sensory modalities. If a subject is highly averse to lotteries when they are presented visually, she would be as averse to lotteries that are presented aurally. Likewise, the consistency of one’s choices is not affected by the modality in which they are presented (Levy and Glimcher, 2011).

Because in our task probabilities and amounts were presented serially we wanted to make sure that the presentation order did not influence subjects’ risk preferences. Therefore, we split the data into trials in which the probability appeared first and to trials in which the amount appeared first and compared the average estimated risk parameter. We did not find an effect for the order of amount and probability presentations, comparing α_probability first_ and α_amount first_ (auditory: *Z* = −0.027, *p* = 0.97; visual: *Z* = −0.3, *p* = 0.76, Wilcoxon rank-sum test).

Finally, we wanted to ensure that any differences in value did not generate differences in RTs. The task was designed in a way that prevents any RTs differences, by introducing a wait period after the presentation of the lottery information and the implementation of the choice. However, any lingering differences in RT could create major confounds in the neural representations and must therefore be addressed. A linear regression of RTs and SVs has revealed no connection between the two (auditory coefficient = −0.004, *p* = 0.49; visual coefficient = −0.008, *p* = 0.5). We therefore conclude that any neural representation of value is not related to RTs. Another concern was that similarity in RTs between modalities would generate a similarity in neural representation that is not directly related to value. To address this issue, we correlated each subject’s RTs to visual lotteries to their RTs to auditory lotteries. The correlation coefficients ranged from −0.24 to 0.28 across subjects, with only three out of the 26 subjects exhibited a correlation of *p* < 0.05, but none were *p* < 0.01. We conclude that similarity in RTs alone cannot explain the similarity in value representation.

### Neuroimaging

#### Common value-representation: GLM

**Figure 4.**
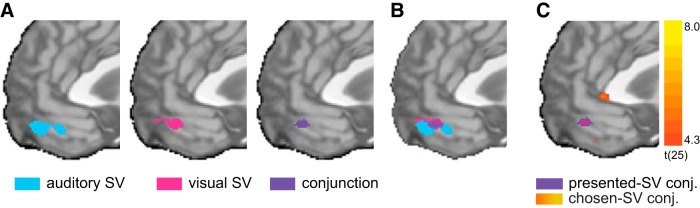
Common value representations across sensory modalities. Whole-brain random-effects maps; *n* = 26. ***A***, Significant voxels tracking aSV, vSV, and a conjunction between the two modality-specific SV predictors (aSV ∩ vSV). ***B***, All three maps superimposed on each other. ***C***, Conjunction between the two modality-specific chosen SV predictors (aCSV ∩ vCSV), superimposed on the presented subjective-value conjunction map. All maps are shown at MNI coordinate *x* = 0. All maps are at *p* < 0.05 (FDR corrected) but shown in different thresholds for presentation purposes (aSV at *z* = 6.5, vSV at *z* = 4, conjunction at *z* = 4.3).

To identify areas of the brain which represent value across sensory modalities, we looked for voxels sensitive to SV of options presented aurally (*aSV*) or visually (*vSV*). To this end, we used the estimated modality-specific individual risk-preferences to calculate each trial’s SV, by raising the lottery’s amount to the power of α and multiplying it by the winning probability (probability × amount^α^; see Materials and Methods). We used the trial-by-trial SV variation in a GLM, with a separate predictor for each modality (*aSV* and *vSV*). As can be seen in Figure 4*A*, contrasting *aSV* with baseline revealed significant activations (q(FDR) < 0.05) in the vmPFC (MNI coordinate: 1, 46, −16). Contrasting *vSV* against baseline also revealed significant positive activations (q(FDR) < 0.05) in the vmPFC (MNI coordinate: 1, 47, −13).

Next, to identify voxels that represent value in both modalities concurrently, we constructed a conjunction analysis of both conditions (*aSV* ∩ *vSV*). As can be seen in Figure 4*A*, we found that activations in an anterior part of the vmPFC (MNI coordinate: 0, 46, −14) positively tracks SV for both sensory modalities.

Note, that in our analysis we modeled the presented-SV, that is, the SV of lotteries, irrespective of subject’s choice. Although the presented-SV and the chosen-SV are highly correlated (mean R across subjects and runs = 0.81, SD = 0.18), we looked for value representation of the chosen alternative and not only the offer value (Raghuraman and Padoa-Schioppa, 2014), for completeness. We constructed another GLM with chosen-SV as our main predictor instead of presented-SV. We found a highly similar neural response to our previous GLM. A conjunction analysis of *aSV_chosen_* ∩ *vSV_chosen_* revealed a cluster in the vmPFC, located at the MNI coordinate: 0, 45, −14, overlapping with the cluster identified for presented-SV (Fig. 4*C*).

#### Common value-representation: MVPA

To further examine the neural substrate of SV and how modality influences it, we turned to multivariate pattern analysis (MVPA) using a cross-modality algorithm. This analysis can show that the value representations not only coincide in a similar anatomic region but are in fact functionally interchangeable. For each subject, we split the trials of each sensory modality into high- and low-value conditions, based on the median SV. This resulted in four conditions: auditory-high, auditory-low, visual-high, and visual-low. We extracted the BOLD signal from the vmPFC, and conducted a cross-modality classification analysis. We trained a SVM to classify between high- and low-value trials based on the pattern of activity of the vmPFC in one sensory modality and then tested the model on the trials of the other sensory modality, and used a permutation test for significance (see Methods). We considered voxels that significantly classify the trials to represent value in a modality-free manner, since they are sensitive to the difference between high and low values but are not sensitive to the sensory modality in which the lotteries were presented.

**Figure 5.**
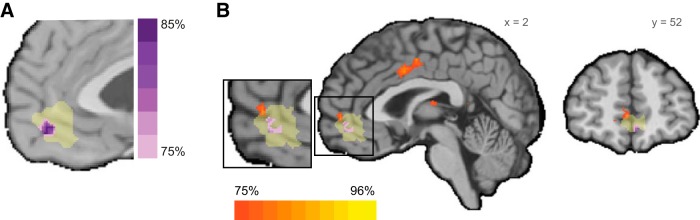
Cross-modality classification results; *n* = 26. To establish a common representation across sensory modalities, we conducted a cross-modality classification analysis. A SVM trained to classify high- from low-value trials of one sensory modality and tested on the other. Hence, significant voxels are sensitive to value and not sensitive to sensory modality. ***A***, Results from a vmPFC-only analysis. A probability map for a voxel to be significant across subjects, at a threshold of 75% and up (in purple). In yellow, the area of the vmPFC mask. The map is shown at MNI coordinate *x* = −3. ***B***, Results from a whole-brain analysis (in orange), superimposed on the vmPFC-only result (in purple).

In significant voxels, we found an average classification accuracy across subjects of 59% (SD: 1.17%, range: 56.94–62.24%). To identify where in the vmPFC this common representation of value is located, we created a probability map across subjects. We counted the number of subjects that a given voxel was significant in their analysis. Figure 5*A* shows the resulting probability map of the vmPFC when we set the presented voxels to a minimum of 75% of subjects. A voxel with a high probability level is a voxel that has a shared representation for *aSV* and *vSV* in many of the subjects. Importantly, the cluster which is common to most subjects (85%) was located in the anterior part of the vmPFC. To ensure the robustness of this result, we also looked at a whole-brain level for voxels that successfully classify value in a cross-modality manner. This analysis revealed a common value representation at the medial PFC, at a slightly more dorsal and anterior location (MNI coordinates 7, 55, −1; for full list of findings, see Fig. 5*B*;Table 1). Both the vmPFC-only and whole-brain findings are in accordance with previous work (Smith et al., 2010), that identified the anterior vmPFC (aVMPFC; MNI coordinates 0, 46, −8) as a region sensitive to experienced value. More specifically, the authors report a conjunction result of monetary value and social value, located at a strikingly similar area of the vmPFC as the result we report in the GLM analysis (MNI coordinate 0, 46, −14) as well as in the vmPFC-only MVPA analysis (MNI coordinate −3, 48, −10).

**Table 1.** Whole-brain cross-modality classification results

**ROI**	**MNI coordinates**			**Number of voxels**	**Average classification accuracy, SD**	**Shared across subjects**
	******x******	******y******	******z******			
L OFC	−39	28	−16	145	59.21%, 1%	96%
L anterior insula	−37	20	−7	129	59.20%, 1%	96%
Thalamus	−9	−25	7	119	59.14%, 0.92%	92%
R amygdala	29	−1	−24	76	59.14%, 1.09%	96%
vmPFC	7	55	−1	43	59.12%, 1.06%	88%
PCC	9	−43	3	47	58.94%, 1.38%	96%
Cerebellum	−7	−49	−9	46	58.77%, 1.13%	88%
Mid-CC	4	−2	39	82	58.74%, 0.86%	96%

Table summarizes details of eight ROIs identified in a whole-brain searchlight MVPA.

#### Value modulation of sensory areas

**Figure 6.**
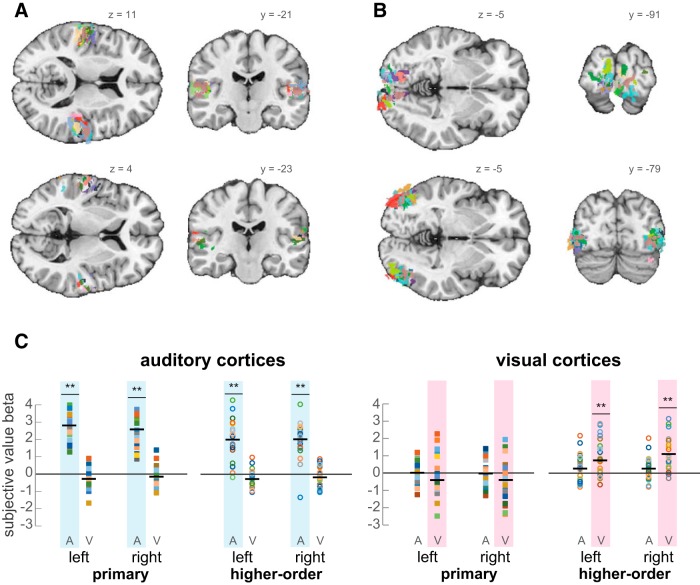
Value modulation of sensory cortices. Individual subjects’ sensory ROIs were defined using functional localizers, and subjective-value β-values were extracted and tested against zero. ***A***, ***B***, For each subject, four contrasts were defined to identify primary and higher-order areas of each modality. Each color represents an individual subject. Upper panels. Primary auditory (***A***) and visual (***B***) cortices. Lower panels, Higher-order auditory (***A***) and visual (***B***) cortices. ***C***, SV representation in these areas. The *y*-axis represents the extracted β-values from a GLM, which included four predictors: aSV (denoted A), vSV (denoted V), and two dummy variables for trial (results not shown here). Each colored marker represents an individual-subject’s β-value. Black horizontal lines represent the means; **p < 0.001, Bonferroni corrected.

Next, we focused on the sensory cortices themselves and examined whether they too convey value information and if the neural representation is specific to a sensory modality. By presenting a series of sounds and images while scanning subjects’ neural activity (see Materials and Methods, Functional localizers), we identified the primary and higher-order visual and auditory cortices for each subject. We defined eight functional ROIs for each subject: right and left primary auditory cortex, right and left higher-order auditory cortex (found at the superior temporal gyrus), right and left primary visual cortex, and right and left higher-order visual cortex [the lateral occipital cortex (LOC); Fig. 6*A*,*B*]. We then used the same GLM as in the common value-representation analysis. This univariate model holds four main predictors: two dummy variables for modality (*aStick*, *vStick*), and two subjective-value predictors, separated by modality (*aSV*, *vSV*), which represent the subject-specific trial-by-trial variation of SV. For each subject, we extracted the β-values for *aSV* and *vSV* predictors from each of the eight ROIs, and tested if it tracks SV (i.e., significantly different from zero).

As can be seen in Figure 6*C*, we found that the visual and auditory cortices indeed represent SV, but they do so in a modality-specific way. That is, unlike the vmPFC which represents value irrespective of the modality in which the information is presented to the subject, the sensory cortices represent value only for their corresponding sensory modality. Interestingly, we found that whereas all four subject-specific auditory ROIs are significantly influenced by SV (maximal *p* < 0.0001, Bonferroni corrected, two-tailed *t* test), only the higher-order visual cortices are sensitive to changes in SV (maximal *p* < 0.0001, Bonferroni corrected, two-tailed *t* test). To ensure the specificity of the effect, we have directly compared the regression coefficients (β-values) between modalities, using a paired *t* test. We confirmed our original analysis, showing that *aSV* is significantly greater than *vSV* in all four auditory ROIs (all *p* < 0.0001), while *vSV* is greater than *aSV* in only the left and right higher-order visual ROIs (*p* = 0.028 and *p* = 0.0003, respectively, FDR corrected).

To ensure that the auditory cortices were properly defined, we repeated this analysis using an alternative method of defining the ROIs. We downloaded two maps from the web-based tool NeuroSynth (Yarkoni, 2014), corresponding to the search-terms Heschl gyrus and planum temporale (primary and higher-order auditory cortices, respectively). We set both maps to a threshold of *z* = 10.5 to maximize the spatial separation between the maps and applied the same GLM from the original analyses and extracted the β-values of *aSV* and *vSV*. We repeated the two approaches to test for the value representation in these ROIs. First, we compared the β-values to zero using a one-sample *t* test, and second, we directly compared *aSV* to *vSV*. This meta-analytical approach to define the ROIs has replicated the results we obtained with the functional localizer, namely, all four ROIs show a significant representation of *aSV*, but not *vSV* (all *p* < 0.0001, Bonferroni corrected).

Finally, to assert that the ROIs were defined properly and hold sensory information, we statistically compared the β-values of *aStick* and *vStick* to zero. We found that each sensory ROI is active in a modality-specific manner. That is, primary and higher-order visual regions responded to visual trials but not to auditory trials, whereas primary and higher-order auditory regions responded to auditory trials but not to visual ones (visual ROIs: *vStick* mean activity = 0.62, mean SD = 0.46, all *p* < 0.001; *aStick* mean activity = 0.007, mean SD = 0.27, all *p* values nonsignificant; auditory ROIs: *aStick* mean activity = 0.56, mean SD = 0.4, all *p* < 0.005; *vStick* mean activity = 0.04, mean SD = 0.27, all *p* values nonsignificant).

## Discussion

The results of our study provide novel insights into the nature of the human *common currency network*. Using a within-subject design, we directly compared the behavioral and neural representations of systematically and rigorously measured SVs of two sensory modalities. On a behavioral level, there were no differences, on average, in subjects’ attitude toward risk when comparing visual and auditory lotteries. In fact, individual’s risk-preferences were highly correlated between modalities. On the neural level, we found that the anterior portion of the vmPFC tracks SV irrespective of the sensory modality in which choice alternatives are presented. We demonstrate this using both a univariate approach, by creating a conjunction analysis of trial-variations in SV, as well as with a cross-modality classification algorithm, which showed some voxels of the vmPFC to be sensitive to value but not to modality. Finally, we show that visual and auditory cortices, defined functionally for each subject, are also sensitive to value. Unlike the vmPFC value region, the sensory cortices hold value information in a modality-specific manner, i.e., the visual cortex is sensitive to the value of lotteries represented visually, while the auditory cortex is sensitive to the value of lotteries represented aurally.

Our main finding extends the notion that the vmPFC is part of a unified value-system of the brain that represents value on a common scale. This common representation has been shown previously to span many features of the decision-making process (Levy and Glimcher, 2012). Evidence have accumulated to show that the activity of the vmPFC is agnostic to rewards’ category (e.g., food, money, social rewards, etc.; Chib et al., 2009; Levy and Glimcher, 2012), to whether the reward is associated with a stimulus or an action (Gläscher et al., 2009), as well as to the phase of the decision process (Clithero and Rangel, 2014). Even at the outcome level, it seems that the vmPFC represents value irrespective of the receiver of the reward (Janowski et al., 2013). The present study broadens our understanding of the common currency network to include the sensory domain, showing value representation irrespective of modality.

The loci of activity which we report here are remarkably similar to the ones reported by other researchers investigating the properties of the common currency network (Chib et al., 2009; Smith et al., 2010; Levy and Glimcher, 2011). In past years, a growing body of evidence has raised the idea that the vmPFC is divided into subregions, some of which represent value in an abstract manner, while others are sensitive to reward category (McNamee et al., 2013; Clithero and Rangel, 2014) and to the type of value, experienced versus goal directed (Smith et al., 2010). Our results suggest that in the anterior vmPFC resides a more abstract and common representation of value, in support of the posterior-to-anterior gradient which corresponds to the concrete-to-abstract representation (Clithero and Rangel, 2014).

Our second finding is that visual and auditory cortices, defined functionally for each subject, are also sensitive to value. Previous studies have hinted to this fact. For example, a study has shown that the activity of the face-selective region of the visual cortex (FFA) is correlated with subjective ratings of the attractiveness of faces (Chatterjee et al., 2009). Likewise, pleasant music has shown to activate the auditory cortex more than music rated as less pleasing (Mueller et al., 2015). Another study found the somatosensory cortex to encode reward delivery, during a somatosensory-discrimination task (Pleger et al., 2008). Notwithstanding, our dual-modality within-subject design enabled us to better examine the extent of this representation and to further characterize it. First, we found that the representation in the sensory cortices is modality specific. That is, the visual cortex represents value of visual but not auditory stimuli, and vice versa. Second, we found evidence for a discrepancy between the visual and auditory cortices in respect to the hierarchy of representation. Whereas both primary and secondary auditory cortices exhibit sensitivity to value, only the secondary visual cortex, namely, the LOC, shows such an activity. We hypothesize that this relates to the inherent difference in the processing streams of auditory and visual information. It has been previously suggested that the primary auditory cortex is not equivalent to the primary visual cortex, but to a more higher-order region (King and Nelken, 2009). This claim corresponds with our results, by suggesting that value representation, as measured in our task and with the limitations of fMRI analyses, is restricted to higher-order regions of the sensory cortices.

Value representations in sensory areas suggest that value representations are evident throughout the brain and are not confined only to higher cognitive areas. It raises interesting questions that need to be addressed in future studies. First, is value a property of a stimulus, much like color or location? And if so, is every sensory representation “tagged” with value? It seems that valuation occurs without overt choices (Levy et al., 2011; Smith et al., 2014), and even when subjects’ attention is drawn toward nonreward properties of a stimulus (Lebreton et al., 2009). Hence, it is plausible that value is an inherent property of the sensory experience. However, a stimulus’ value changes in response to learning and experience. This suggest that value may be modulating the representation of a stimulus, perhaps like attention (Moran and Desimone, 1985). Some previous work has proposed a mechanism in which sensory information is conveyed from sensory cortices into the OFC, where identity-specific value information is formed, alongside an identity-general value representation in the vmPFC. The connectivity between the OFC and the relevant associative cortex (piriform cortex, in the olfactory case) depends on the value of the stimulus (Howard et al., 2015), and its connectivity with the vmPFC changes as the value changes, e.g., when a subject becomes sated with a specific odor and its value declines (Howard and Kahnt, 2017). Naturally, the sensory representation of the sated odor must not change, but the input to the value system does. This raises another open question: how does value information travel between sensory and value regions, is value inferred within the sensory network and then transferred to the prefrontal cortex to facilitate the decision-making process, in a bottom-up manner? Or is it the other way around, with sensory information arriving to the prefrontal cortex, there tagged with value and fed back to sensory areas, in a top-down process? Some previous work has been done on the issue, by using electroencephalography (EEG) to record the dynamics of value processing in the brain (Harris et al., 2011). Harris and colleagues found value information as early as 100–150 ms after stimulus onset in visual areas (namely, the lingual gyrus). Moreover, they show a potential causal link between these early visual areas and activity in the vmPFC, with an opposite-direction link on a later timepoint. Hence, decision making may involve an iterative process between the valuation and sensory systems. However, additional work is still needed, employing tools which are better suited for answering questions of directionality, such as transcranial magnetic stimulation (TMS) or electrophysiology of single units. Such tools can directly examine the causal link between sensory and value information. Finally, another relevant question is how low in the sensory domain can we go down and still observe value tagging? There is evidence that already at the receptor level there is adaptation and a Weber–Fechner-like transformation of the sensory information (Stevens, 1970), which seem to contradict our finding that the early visual cortex does not show value information. The temporal imprecision of the fMRI renders it unsuitable to definitively answer this question, and more studies need to be conducted with higher temporal and spatial resolutions to examine this question further.

As the TR in our design was an integer multiple of the ITI, this could create potential over- or underestimation of differences in neural activity across slices of the brain and pose a limitation for our findings. However, this limitation does not affect the main finding we report here, which involves comparison of neural activity in the same exact region (vmPFC), and not across slices. The only finding that might be affected by the biased sampling of the HRF is the discrepancy we find between primary and high-order visual cortices in their representation of value. In this case, both areas are located in slices close to each other, in respect to the sampling sequence (mean *z* MNI coordinate, primary visual cortex = −3, higher-order visual cortex = −4), rendering any potential bias minimal.

In summary, we show that the common currency network of the human brain represents the value of stimuli irrespective of the sensory domain in which they were presented. Additionally, we show that sensory cortices hold information regarding the value of stimuli in a modality-specific manner. These findings bring our understanding of the neural valuation system a step closer to real-world environments, where individuals choose between multi-dimensional alternatives, composed of information from different domains and sensory modalities.

**Table 2.** Statistical table

	Data structure	Type of test	Power
a (α across modalities)	Fitted data, non-normal	Rank sum	CI of auditory-α: 0.52–0.719CI of visual-α: 0.52–0.709CI of difference: −0.01–0.09
b (α correlation)	Fitted data, non-normal	Spearman’s correlation	CI of *r*: 0.973–0.9755
c (β correlation)	Fitted data, non-normal	Spearman’s correlation	CI of *r*: 0.9453–0.9508
d (order effect, auditory)	Fitted data, non-normal	Rank sum	CI of amount-first-αs: 0.529–0.72CI of probability-first-αs: 0.52–0.71CI of difference: −0.01–0.01
d (order effect, visual)	Fitted data, non-normal	Rank sum	CI of amount-first-αs: 0.52–0.7CI of probability-first-αs: 0.53–0.71CI of difference: −0.02–0.004
e (RTs and value)	Fitted data, non-normal	Linear regression	Auditory RT coefficient CI: −0.016–0.008Visual RT coefficient CI: −0.035–0.017
f (neuroimaging conjunction)	Normal distribution	*t* test	aSV β-value CI: 0.43–0.71vSV β-value CI: 0.25–0.56
g (chosen-SV presented-SV correlation)	Normal distribution	Pearson correlation	CI of *r*: 0.74–0.87
h (chosen-SV neuroimaging conjunction)	Normal distribution	*t* test	aSV β-value CI: 0.48–0.805vSV β-value CI: 0.27–0.60
i (MVPA accuracy)	Normal distribution	Permutation test (per subject)	CI: 54.5–63.49% accuracy
j (whole-brain MVPA accuracy, vmPFC)	Normal distribution	Permutation test (per subject)	CI: 58.68–59.55% accuracy
k (sensory ROIs value representation)	Normal distribution	One-sample *t* tests	Primary left auditory cortex, aSV: 2.3–3.31Primary right auditory cortex, aSV: 2.01–3.14Higher-order left auditory cortex, aSV: 1.28–2.75Higher-order right auditory cortex, aSV: 1.39–2.68Primary left visual cortex, aSV: −0.44–0.47Primary right visual cortex, aSV: −0.46–0.31Higher-order left visual cortex, aSV: −0.17–0.68Higher-order right visual cortex, aSV: −0.12–0.63Primary left auditory cortex, vSV: −0.59–0.03Primary right auditory cortex, vSV: −0.48–0.17Higher-order left auditory cortex, vSV: −0.58–0.07Higher-order right auditory cortex, vSV: −0.5–0.19Primary left visual cortex, vSV: −1.3–0.45Primary right visual cortex, vSV: −1.15–0.29Higher-order left visual cortex, vSV: 0.11–1.28Higher-order right visual cortex, vSV: 0.5–1.55
l (sensory ROIs value representation, between-modality tests)	Normal distribution	Paired *t* tests (one tailed)	Primary left auditory cortex, aSV > vSV: 2.7-infPrimary right auditory cortex, aSV > vSV: 2.3-infHigher-order left auditory cortex, aSV > vSV: 1.8-infHigher-order right auditory cortex, aSV > vSV: 1.78-infPrimary left visual cortex, vSV > aSV: -inf-0.9Primary right visual cortex, vSV > aSV: -inf-0.8Higher-order left visual cortex, vSV > aSV: inf-(−0.06)Higher-order right visual cortex, vSV > aSV: -inf-(−0.44)
m (sensory ROIs sensory information)	Normal distribution	One-sample *t* tests	Primary left auditory cortex, aSt: 0.3–0.82Primary right auditory cortex, aSt: 0.3–0.83Higher-order left auditory cortex, aSt: 0.23–0.75Higher-order right auditory cortex, aSt: 0.42–0.86Primary left visual cortex, aSt: −0.06–0.25Primary right visual cortex, aSt: −0.05–0.34Higher-order left visual cortex, aSt: −0.28–0.02Higher-order right visual cortex, aSt: −0.25–0.09Primary left auditory cortex, vSt: −0.06–0.24Primary right auditory cortex, vSt: −0.16–0.13Higher-order left auditory cortex, vSt: −0.14–0.3Higher-order right auditory cortex, vSt: −0.11–0.2Primary left visual cortex, vSt: 0.29–0.98Primary right visual cortex, vSt: 0.55–1.12Higher-order left visual cortex, vSt: 0.19–0.75Higher-order right visual cortex, vSt: 0.24–0.81
n (anatomically defined auditory ROIs value information)	Normal distribution	One-sample *t* tests	Hescel left, aSV: 2.6–3.2Hescel right, aSV: 2.7–3.4Planum temporale left, aSV: 2.1–2.8Planum temporale right, aSV: 1.4–2.3Hescel left, vSV: −0.3–0Hescel right, vSV: −0.3–0.06Planum temporale left, vSV: −0.3–0.01Planum temporale right, vSV: −0.43–0.07
o (anatomically defined auditory ROIs value information, between-modality tests)	Normal distribution	Paired *t* tests (one tailed)	Primary left auditory cortex, aSV > vSV: 2.8-infPrimary right auditory cortex, aSV > vSV: 2.9-infHigher-order left auditory cortex, aSV > vSV: 2.3-infHigher-order right auditory cortex, aSV > vSV: 1.6-inf

Table summarizes the distribution, statistical test, and power for each analysis in this study.

## Synthesis

Reviewing Editor: Daniela Schiller, Mount Sinai School of Medicine Decisions are customarily a result of the Reviewing Editor and the peer reviewers coming together and discussing their recommendations until a consensus is reached. When revisions are invited, a fact-based synthesis statement explaining their decision and outlining what is needed to prepare a revision will be listed below. The following reviewer(s) agreed to reveal their identity: Vikram Chib.

The degree to which value signals are modality-specific is a timely issue. The paper is well written and has the potential to make a relevant contribution to the literature by showing modality-independent value signals both with univariate and multivariate analyses and modality-specific value signals in primary and higher auditory and visual areas.

The study examined how regions such as the ventromedial prefrontal cortex and primary and secondary auditory and visual cortices process subjective value when decisions options are presented in auditory or visual modality. Both univariate and multivariate analyses show modality-independent value coding in ventromedial prefrontal cortex and modality-dependent value coding in sensory areas.

The authors have addressed the points raised by the previous reviewers well, with the one small exception that reviewer 2 asked for a reference for the specific random utility model used (point 6). Nevertheless, there are several points that should be addressed before the paper goes ahead.

Major:

1. It is unclear whether cross-modality decoding works because of average differences in response times. Presumably participants were faster in high-value than in low-value trials and these differences held in both modalities. By extension, successful cross-modality decoding could reflect motor confounds. The authors should describe response times and how the neural findings change if they control for response times.

2. TR was 2s and the within-trial timings seem to have been run predominantly in multiples of 2s. If event onsets were not jittered with regard to scan onsets, the design may have sampled the HRF in a biased way, which could have led to wrong inferences, raising doubts on the conclusions that can be drawn from the present research (see e.g., Price, Veltman, Ashburner, Josephs, Friston, 1999, Neuroimage).

3. Throughout, it is unclear whether parametric modulators modeled subjective value as presented (irrespective of choice) or as chosen. The two will be highly correlated but given the emphasis the authors put on ratings not necessarily being reflective of choice one would expect them to model chosen rather than presented subjective value (please specify), which then raises the question of how missed trials were treated in the analysis?

4. In order to claim specificity, the auditory and visual value signals should be compared directly. In other words, was there a significant difference between modalities, such that visually presented value signals were significantly stronger in visual cortex than auditory value signals and vice versa for auditory cortex?

5. The identification of primary sensory regions is not matched for visual and auditory modalities, with the former comparing two different types of visual inputs and the latter comparing auditory input versus no auditory input. Can the authors provide a rationale for the difference and some evidence that the findings are independent of it, e.g., by using functional ROIs from previous meta-analyses? This point is also relevant because of the apparent difference between the auditory (no primary region as such, value signals in both secondary and primary auditory areas) and visual (value signals only in secondary areas) systems highlighted in the discussion.

6. The authors report multivariate data only for ventromedial prefrontal cortex. However, whole-brain analysis would be more informative, even if it is only in the form of a table within the main paper as it would allow to calibrate the strength of the ventromedial findings with regard to other regions.

Minor:

1. The authors did not use sparse imaging, which is fine. However, please specify the make and model of headphones used, the intensity of auditory stimulation (in db) and whether any noise cancellation was applied to ensure understanding of auditory stimuli.

2. Line 114: There appears to be a discrepancy with figure 1 in that the text indicates the choice window to last for 1.7 seconds and the figure for 1.5 seconds. Please clarify.

3. Line 117: 2 seconds sound relatively long for the auditory stimulus presentation. Please specify what exactly was said and whether the voice sounded female or male.

4. Line 123: The details of the 13 options used should be provided in the text or as a table.

5. Lines 136ff: The average (min, max) time period between first and second behavioral session and between second behavioral session and fMRI session should be provided.

6. Line 184: Were data smoothed for both univariate and multivariate analyses (for the latter no smoothing should be used)?

7. Line 185: Please specify how many runs had to be excluded due to movement of more than 3 mm.

8. Lines 290/291, please report also whether beta differed significantly between modalities?

9. Lines 313 (see also line 315) states that several regions, including bilateral auditory cortex can be seen in Figures 4a-b. Only ventromedial prefrontal cortex is shown though. Please reword or adapt figure.

10. Lines 344ff: Please specify more explicitly that the data are from the univariate analysis and describe the meaning of the colors in the legend to figure 6.

11. Lines 446ff: Please take your own data more seriously when discussing how low in the sensory domain we can go to still observe value tagging. On the one hand, you do not observe value tagging in the primary visual cortex, on the other hand, some subjects showed risk seeking rather than risk aversion which is incompatible with Weber-Fechner-like adaptation. Both of these findings suggest that we can not go super low.

Wording:

1. Line 86: could represents -> could represent

2. Line 99: add verb (e.g., ‘could not be analyzed’) between ‘... opted out, 2’ and ‘due to’; also, please specify the the technical problems that led to the exclusion of these subjects

3. Lines 113-114: add ‘was’ between ‘but’ and ‘counterbalanced’

4. Line 292: figure 3b -> Figure 3b

5. Lines 442-443: “Hence, possibly the decision-making processes involves an iterative process” -> “Hence, decision making may involve an iterative process”

6. Line 448: ‘weber-fechner’ -> ‘Weber-Fechner’
